# Correlation of serum IL-2 and IFN-γ levels with clinical prognosis of nasopharyngeal carcinoma patients and analysis of risk factors

**DOI:** 10.5937/jomb0-44057

**Published:** 2024-01-25

**Authors:** Siquan Guo, Feng Qin, Jiang Wang, Yongqing Ding, Jianqiang You, Changjiang Chao

**Affiliations:** 1 Third Affiliated Hospital of Soochow University, Department of Otorhinolaryngology Head and Neck Surgery, Changzhou, China; 2 Nanjing Medical University, Changzhou Third People's Hospital, Changzhou Medical Center, Department of Otorhinolaryngology Head and Neck Surgery, Changzhou, China; 3 First Affiliated Hospital of Hebei North University, Department of Otorhinolaryngology Head and Neck Surgery, Zhangjiakou, China

**Keywords:** IL-2, IFN-γ, nasopharyngeal carcinoma, correlation, risk factors, IL-2, IFN-γ, karcinom nazofarinksa, korelacija, faktori rizika

## Abstract

**Background:**

This study aims to investigate the correlation between serum levels of interleukin-2 (IL-2) and interferong (IFN-g) and the clinical prognosis of patients with nasopharyngeal carcinoma (NPC). Additionally, the study aims to analyse the risk factors associated with this correlation.

**Methods:**

The clinical data of 195 NPC patients admitted to our hospital from October 2020 to October 2022 were selected for a retrospective study. Based on the Glasgow score, patients were divided into two groups: the good prognosis group (group g), consisting of patients who scored 0 points, and the poor prognosis group (group p), consisting of patients who scored 1-2 points. The levels of serum IL-2 and IFN-g were compared between the two groups, and the clinical values of serum IL-2 and IFN-g in the prognosis of patients were analysed. The clinical parameters of the patients were collected, and the risk factors affecting the prognosis of NPC were analysed by univariate and multivariate logistic regression.

## Abstract (sr)

**Uvod:**

Ova studija ima za cilj istraživanje korelacije između nivoa serumskog interleukina-2 (IL-2) i interferon-gama (IFN-g) i kliničke prognoze pacijenata sa karcinomom nazofarinksa (NPC). Takođe, studija ima za cilj analizu rizičnih faktora povezanih sa ovom korelacijom.

**Metode:**

Za retrospektivnu studiju su odabrani klinički podaci od 195 pacijenata sa karcinomom nazofarinksa (NPC) primljenih u našu bolnicu od oktobra 2020. do oktobra 2022. Na osnovu Glasgow skora, pacijenti su podeljeni u dve grupe: grupa sa dobrom prognozom (grupa g), koja je obuhvatala pacijente koji su imali 0 poena, i grupa sa lošom prognozom (grupa p), koja je obuhvatala pacijente sa 1-2 poena. Uporedili smo nivoe seruma IL-2 i IFN-g između ove dve grupe i analizirali kliničku vrednost seruma IL-2 i IFN-g u prognozi pacijenata. Prikupljeni su klinički parametri pacijenata, a faktori rizika koji utiču na prognozu NPC su analizirani univarijatnom i multivarijatnom logističkom regresijom.

**Rezultati:**

Od 195 pacijenata, 125 je bilo u grupi g, a 70 u grupi p. Nivoi IL-2 i IFN-g u grupi g su bili viši nego u grupi p (P<0,05). Prediktivni AUC (95%CI) za IL-2 i IFN-g su iznosili 0,846 (0,791-0,902) i 0,851 (0,797-0,904), redom (P<0,05). Analiza logističke regresije je pokazala da su faktori rizika za prognozu pacijenata sa NPC-om starost, prečnik tumora, klinički stadijumi i stepen diferencijacije.

**Zaključak:**

Nivoi seruma IL-2 i IFN-g su tesno povezani sa prognozom pacijenata sa NPC-om i imaju određenu vrednost u proceni prognoze pacijenata. Starost, prečnik tumora, stadijumi tumora i stepen diferencijacije tumora su svi faktori rizika koji utiču na prognozu pacijenata, što može biti referenca za odabir kliničkih metoda lečenja i procenu prognoze.

## Introduction

Nasopharyngeal carcinoma (NPC) refers to cancer that occurs in the nasopharynx, whose principal symptoms include nasal mucus with blood, nasal obstruction, tinnitus and deafness, jugular malignant nodule and headache, as one of the tumours with high incidence in China [1]. According to statistics [2], NPC accounts for 60% of malignant tumours in the otorhinolaryngology department, and the incidence of males is 2-3 times that of females. NPC mostly occurs in the posterior wall of the nasopharynx and the pharyngeal recess, which has a low diagnosis rate in the early stage due to the hidden location and fewer early features, and it is often in the middle and late stages when diagnosed, missing the best treatment time. The NPC seriously affects patients' prognosis and quality of life because of low differentiation degree, high malignant degree and easy occurrence of cancer metastasis. In recent years, there have been more and more discussions on the correlation of inflammation with tumours. When the body is damaged or invaded by pathogens, the immune system activates and recruits many inflammatory cells for infiltration to secrete multiple cytokines, exerting a vital role in the occurrence and development of tumour cells [3] [4]. Serum interleukin-2 (IL-2), a cytokine with wide biological activity, plays a crucial role in anti-viral and antineoplastic activities, enhances immune function, and participates in multiple physiological and pathological reactions [5]. Studies have shown that [6] IL-2 can be used as a marker to monitor the diagnosis and treatment of various malignant tumours. Interferon-γ (IFN-γ), a pleiotropic cytokine with anti-viral, antineoplastic and immunomodulatory properties, activates mononuclear phagocytes to kill microorganisms and cytotoxic T lymphocytes to kill infected target cells [7]. Researchers have found that [8] serum IL-18 level is associated with the prognosis of NPC patients. However, there are few studies on the correlation of IL-2 and IFN-γ levels with the prognosis of NPC patients. Therefore, this study used IL-2 and IFN-γ as inflammatory indicators to explore the correlation with the prognosis of NPC patients and analysed the risk factors of prognosis to provide more references for clinical practice, with the report summarised as follows.

## Materials and methods

### Study design

The clinical data of 195 NPC patients admitted to our hospital from October 2020 to October 2022 were selected for a retrospective study to explore the correlation of serum levels of IL-2 and IFN-γ with their clinical prognosis and analyse the risk factors.

### Inclusion and exclusion criteria

*Inclusion criteria:* (1) The patients diagnosed with NPC via imaging, cytology, microscopy, and pathology met the diagnostic criteria of NPC in Experts Consensus on Comprehensive Treatment of Head and Neck Cancer [9]. (2) Patients had no other disease in the nose and throat. (3) Patients had complete clinical data. (4) The predicted survival time was more than 6 months. (5) Patients had normal cognitive function. (6) Patients and their families who knew the purpose and process signed the informed consent. (7) This study was in line with the Declaration of Helsinki (2013) [10].

*Exclusion criteria*: (1) Patients with severe dysfunctions in the heart, liver and kidney; (2) patients who could not communicate with others due to mental diseases; (3) patients with general immune system diseases; (4) patients who could not tolerate radiotherapy; (5) patients with other malignant tumours; (6) patients with distant metastasis; (7) patients with severe complications; and (8) patients who were in gestation period or lactation period.

## Methods

### Treatment methods

All patients received nasopharyngeal and jugular radical radiotherapy using a linear accelerator with high energy for radiotherapy. The external irradiation of conventional segmentation in the nasopharynx was adopted at a dose range of 66-76 Gy, 1.8-2 Gy/time, and 5 times/week for 6-7.5 weeks. According to the patients' actual residue situation, local shrinkage-field irradiation at a dose of 6-10 Gy was performed. The total cervical lymph node metastasis dose was 64-72 Gy for 6-7 weeks, and the total dose of jugular preventive irradiation was 50-60 Gy for 5-6 weeks.

### Serum detection

Before treatment, 3 mL of fasting peripheral cubital venous blood was taken from patients in the morning to centrifuge at 3000 r/min for 10 min, and the supernatant was taken and placed in a refrigerator at -80°C for testing. Serum IL-2 and IFN-γ levels were measured by enzyme-linked immunosorbent assay. The kit was purchased from Shanghai Enzymelinked Biotechnology Co., Ltd. (Shanghai, China), and all operations were strictly carried out following the instructions with the following steps.

Detecting steps of serum IL-2: (1) The required slats were taken out from the aluminium foil bag after equilibrium with room temperature for 20 min, and the remaining slats were sealed with a valve bag and placed back at 4°C. (2) The standard well and the sample well were set up, and each standard well was added with 50 μL of different concentrations of standards. (3) 50 μL of the sample to be tested was added to the sample well, not the blank well. (4) Except for the blank well, 100 μL of detecting antibody labelled with horseradish peroxidase (HRP) was added to each standard well and sample well. The reaction wells were sealed with the plate seal and incubated in the 37°C water bath or thermostat for 60 min. (5) The liquid was discarded and patted dry on the bibulous paper. The cleaning solution (350 L) was added to each well, removed after standing for 1 min, and then patted dry on the bibulous paper. The plate was washed five times repeatedly (The plate was also washed with a plate washer). (6) 50 μL of substrates A and B were added to per well and incubated blindly at 37°C in the dark for 15 min. (7) The OD value of each well was measured at 450 nm of wavelength within 15 min after adding 50 μL of stopping solution to each well.

Result calculation: The horizontal axis represented the OD value of the measured standard the vertical axis represented the concentration of the standard. The standard curve is drawn on square paper or with the relevant software, and the linear regression equation is obtained. The OD value of the sample is substituted into the equation to calculate the concentration of the sample.

Sensitivity: The lowest detection concentration is <10 pg/mL.

Detecting steps of serum IFN-γ: (1) The required slats were removed from the aluminum foil bag after being equilibrated at room temperature for 20 minutes, and the remaining slats were sealed in a valve bag and placed back at 4. (2) The standard well and the sample well were prepared, and each standard well was added with 50 μL of different concentrations of standards. (3) 50 μL of the test sample was added to the sample well, but not to the blank well. (4) Except for the blank well, 100 μL of detecting antibody labeled with horseradish peroxidase (HRP) was added to each standard well and sample well. The reaction wells were sealed with a plate seal and incubated in a 37°C water bath or thermostat for 60 minutes. (5) The liquid was discarded and gently dried using absorbent paper. Cleaning solution (350 μL) was added to each well, allowed to stand for 1 minute, and then removed by patting dry with absorbent paper. The plate was washed five times (the plate can also be washed with a plate washer). (6) 50 μL of substrates A and B were added to each well and incubated at 37°C in the dark for 15 minutes. (7) Each well's optical density (OD) value was measured at a wavelength of 450 nm within 15 minutes after adding 50 μL of stopping solution to each well.

Result calculation: The OD values of the measured standards were plotted on the x-axis, and the corresponding concentrations of the standards were plotted on the y-axis. The standard curve can be drawn on graph paper or using relevant software, and the linear regression equation can be obtained. The OD value of the sample is substituted into the equation to calculate its concentration.

Sensitivity: The lowest detectable concentration is <10 pg/mL.

### Follow-up visit and observation indices

The patients were followed up for 1 year by outpatient examination, telephone interview and on-site visit. According to the Glasgow score, the patients were divided into the good prognosis group (group g, n=125) and the poor prognosis group (group p, n=70).

The levels of serum IL-2 and IFN-γ before treatment were compared between the two groups. The values of serum IL-2 and IFN-γ in evaluating the prognosis of patients were analysed. Univariate analysis was used to compare the general data of the two groups, including gender, age, BMI, tumour diameter, clinical stages and degree of differentiation. Multivariate logistic regression analysis was performed on factors with statistical differences to explore the risk factors affecting the prognosis of patients.

### Statistical method

The data processing software in this study was SPSS20.0. The enumeration data and measurement data were tested by x^2^ test and t test, expressed as (n (%)) and (x̄±s). The ROC curve was used to analyse the prognostic values of serum IL-2 and IFN-γ levels, and logistic regression analysis was used to explore the influencing factors. The statistical results were P<0.05, indicating statistical differences in this group of data.

## Results

### Comparison of serum levels of IL-2 and IFN-γ in both groups

The levels of IL-2 and IFN-γ in group g were higher than in group p (P<0.05), as shown in Table 1.

**Table 1:** Comparison of serum levels of IL-2 and IFN-γ in both groups (x̄±s).

Groups	Cases	IL-2 (ng/mL)	IFN-γ (ng/L)
Group g	125	5.05±1.00	32.79±3.54
Group p	70	3.80±0.72	27.41±3.28
t		9.177	10.442
P		<0.001	<0.001

### Prognostic values of serum IL-2 and IFN-γ levels in NPC

**Figure 1:**
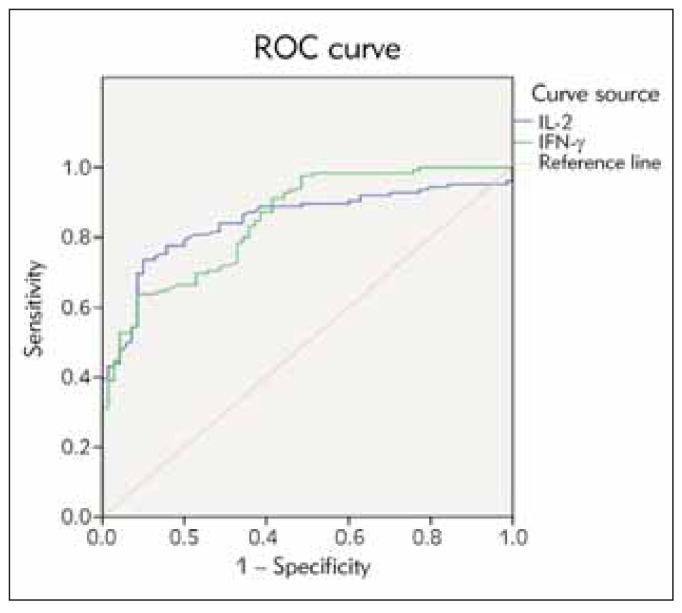
Prognostic values of serum IL-2 and IFN-γ levels inNPC.

ROC showed that the predictive AUC (95%CI) of IL-2 and IFN-γ were 0.846 (0.791-0.902) and 0.851 (0.797-0.904), respectively (P<0.05), as detailed in Figure 1 and Table 2.

**Table 2:** Area under the curve.

Variables<br>of test <br>results	Area	Standard<br>error	Sig	Asymptotic 95%<br>confidence interval	
				Lower limit	Upper
IL-2	0.846	0.028	0.000	0.791	0.902
IFN-g	0.851	0.027	0.000	0.797	0.904

### Univariate analysis of clinical parameters affecting the prognosis of patients

There were distinct differences in age, tumour diameter, clinical stages and degree of differentiation (P<0.05), with no overt difference in gender and BMI (P>0.05). See Table 3 for details.

**Table 3:** Univariate analysis of clinical parameters affecting the prognosis of patients.

Indices	Group g (n=125)	Group p (n=70)	X2/t	P
Gender (male/female)	73/52	42/28	0.047	0.828
Age	49.98±6.67	56.30±8.22	5.829	<0.001
BMI (kg/m^2^)	21.30±1.50	21.68±1.49	1.697	0.091
Tumour diameter			43.678	<0.001
5 cm	35 (28.00)	54 (77.14)		
<5 cm	90 (72.00)	16 (22.86)		
Clinical stages			36.115	<0.001
Stage I-stage II	88 (70.40)	18 (25.71)		
Stage III-stage IV	37 (29.60)	52 (74.29)		
Degree of differentiation			28.069	<0.001
Low differentiation	40 (32.00)	50 (71.43)		
Middle and high differentiation	85 (68.00)	20 (28.57)		

### Multivariate analysis of clinical parameters affecting the prognosis of patients

The logistic regression analysis was performed taking the situations of disease prognosis as the dependent variable and age, tumour diameter, clinical stages and degree of differentiation as independent variables, revealing that age, tumour diameter, clinical stages and degree of differentiation were risk factors for the prognosis of NPC patients. See Table 4 and Table 5.

**Table 4:** Variable assignment.

Variables	
Situations of prognosis	0=good prognosis and 1=poor prognosis
Age	Continuous variable
Tumour diameter	0=<5cm and 1≥5cm
Clinical stages	0=stage I-stage and 1=stage III-stage IV
Degree of differentiation	0=middle and high differentiation and 1=low differentiation

**Table 5:** Multivariate analysis of clinical parameters affecting the prognosis of patients.

Factors	β	S.E.	Wals	df	P	Exp(β)
Age	0.125	0.030	17.782	1	<0.001	1.133
Tumour diameter	1.849	0.426	18.872	1	<0.001	6.355
Clinical stages	1.926	0.435	19.626	1	<0.001	6.864
Degree of differentiation	1.436	0.432	11.023	1	0.001	4.202

## Discussion

NPC is a malignant tumour caused by many factors such as heredity, environment, diet and virus infection, whose incidence is still on the rise according to the study data, with the highest incidence in the population aged 40-60 [11] [12] [13]. Ear-nose symptoms, headache, facial numbness, diplopia and neck masses are the most common clinical symptoms in NPC patients, and they will have complex and changeable symptoms due to different primary sites, sizes, external invasion and metastasis sites, affecting the health and safety of patients [14] [15]. The radiation sensitivity and particular anatomical location of NPC cause extreme sensitivity to radiotherapy, so radiotherapy has become the main treatment. However, the malignant degree and recurrence and metastasis rate of NPC patients are high, so the prognosis and risk factors have become the focus of clinical attention.

A related study [16] has found that inflammatory factors in the tumour microenvironment significantly affect the occurrence, development, invasion, and metastasis of tumours. They also play an indispensable role in NPC patients. Serum IL-2 is an immunomodulator secreted by Th1 cells, whose biological role is mainly to stimulate the proliferation of T cells. This proliferation can result in cytotoxic T cells, natural killer cells, and lymphokine-activated killer cells. It enhances killing activity, promotes lymphocytes to secrete antibodies and interferons, and participates in antibody response, hematopoiesis, and tumour surveillance [17]. Serum IL-2 has a role in anti-bacterial and anti-viral infection and in inhibiting the proliferation and differentiation of tumour cells. Therefore, detecting its level is of great significance in diagnosing and treating diseases.

The literature [18] has shown that the IL-2 level in NPC patients is lower than in healthy people but increases after radiotherapy and chemotherapy. The decrease in IL-2 levels in NPC patients may be due to the inhibition of cellular immunity, the damaged regulation effect of cytokines, and the inability of T and B lymphocytes to exert normal immune regulation. This results in a decreased number, supply, and activity of cells produced by IL-2, ultimately reducing IL-2 levels.

In this study, follow-up results showed differences in IL-2 levels between the two groups, with the IL-2 level in group G higher than in group P (P<0.05). It is suggested that a higher IL-2 level before radiotherapy corresponds to a higher survival rate after treatment. ROC curve analysis showed that the predictive AUC (95% CI) of IL-2 was 0.846 (0.791-0.902), confirming that IL-2 is related to the prognosis of NPC patients and has good prognostic value.

IFN-γ, a soluble dimeric cytokine, is vital in innate and adaptive immunity against viral, cellular, and protozoan infections. Its abnormal expression is closely related to many autoimmune diseases [19]. In the inflammatory environment, IFN-γ triggers the activation of the immune response and stimulates the elimination of pathogens. It plays a role in preventing excessive activation of the immune system and tissue damage, and in the tumour microenvironment, IFN-γ coordinates the balance of pro-tumour and antitumour immunity [20].

IFN-γ, an anti-proliferation agent, achieves its anti-tumour effect through multiple processes. It regulates the expression of cyclin-dependent kinase inhibitor 1 (P21) by activating STAT1 in tumour cells. Additionally, IFN-γ inhibits angiogenesis, impairs the survival of endothelial cell proliferation, and induces tumour interstitial ischemia. Hao Kaifei et al. [21] have shown that IFN-γ is a crucial factor in regulating the radiosensitivity of lung cancer and plays a role in radiotherapy by regulating the body's immune function.

This study showed that the IFN-γ level in group G was higher than in group P (P<0.05), suggesting abnormal expression of IFN-γ in NPC cells, causing the production of obvious immunosuppression, decreased cellular immune function, and increased IFN-γ level after treatment. This helps improve patients' condition and increase the survival rate. The predictive AUC (95% CI) of IFN-g was 0.851 (0.797-0.904), indicating that IFN-γ is related to the prognosis of NPC patients. It holds an important value in predicting the prognosis of such patients and can be used as a vital marker for treatment evaluation. 

Furthermore, the study analysed the risk factors affecting the prognosis of NPC patients. Univariate analysis of clinical indicators showed significant differences in age, tumour diameter, clinical stages, and degree of differentiation in both groups (P<0.05). Further multivariate logistic regression analysis revealed that age, tumour diameter, clinical stages, and degree of differentiation were risk factors affecting the prognosis of NPC patients.

Currently, there are relatively few clinical analyses on the influence of age on the prognosis of NPC patients. Some scholars [22] have shown that young patients have better local control and survival rates than elderly patients. This study also demonstrated a specific relationship between age and prognosis; further exploration of its mechanism is warranted.

Tumour diameter, tumour stage, and degree of differentiation can reflect the biological characteristics and development of the tumour. The tumour stage and degree of differentiation are related to the prognosis in many solid tumours. Patients in the late stage with a low differentiation degree generally have a relatively poor prognosis [23].

Tumour stages are determined by factors of the primary tumour, including size, depth of invasion, the extent of involvement of adjacent organs, and presence or absence of local and distant metastasis. They reflect the degree of invasion and metastasis in tumours. Evaluating the extent of invasion and metastasis, the degree of disease progression, and the prognostic indexes of malignant tumours can provide an accurate basis for clinicians to manage patients hierarchically. This is the basic premise for selecting auxiliary treatment and improving therapeutic effect [24].

Tumour differentiation is a process in which tumour cells gradually evolve and mature, reflecting different degrees of morphological differences between tumour tissues and normal tissues regarding tissue structure and cell morphology. In the clinic, the best-differentiated and worst-differentiated regions are usually used to determine the tumour's tissue source and the tumour's grade, respectively. Therefore, the differentiation degree of malignant tumours is an essential reference for reflecting tumours' internal characteristics, evaluating biological behaviour and predicting the prognosis [25].

In summary, serum IL-2 and IFN-γ levels are closely related to the prognosis of NPC patients and have particular value in evaluating the prognosis of patients. The age, tumour diameter, tumour stages and degree of tumour differentiation are all risk factors affecting the prognosis of patients, which can provide a reference for selecting clinical treatment methods and evaluating prognosis.

## Dodatak

### Contribution

Siquan Guo and Feng Qin contributed equally.

### Conflict of interest statement

All the authors declare that they have no conflict of interest in this work.
